# Measles outbreak investigation in Aweil East county, South Sudan

**DOI:** 10.11604/pamj.2021.40.87.28370

**Published:** 2021-10-11

**Authors:** Evans Nyasimi Mokaya, Zingbondo Isaac, Nathan Atem Anyuon

**Affiliations:** 1 African Field Epidemiology Network, Nairobi, Kenya,; 2 Ministry of Health, Aweil, South Sudan

**Keywords:** Measles outbreak, South Sudan, vaccination, outbreak reactive vaccination

## Abstract

During January 2018-June 2020, Aweil East confirmed five measles outbreaks. In March 2020, Aweil East reported twenty measles IgM+ cases. Before this outbreak, Aweil East had confirmed an outbreak in late November 2019. Even after conducting outbreak reactive vaccinations (ORV) in December 2019 and February 2020, measles spread was not interrupted. The nationally supported measles follow-up campaign (MFUC) conducted in late February 2020 was deferred in Aweil East because of the February ORV. We reviewed the measles data collected through passive and active surveillance. A matched case-control study was conducted to evaluate potential exposures. Face-to-face interviews with cases and controls using a semi-structured questionnaire were used to collect demographics, disease, and exposures related data. A total of 687 cases with eight deaths; attack and case fatality rate of 123/100,000 population and 1.16%, respectively. Among the cases, 51.8% were male, the median age was four years, and 59% of cases ≥9 months were unvaccinated. Eighty point six percent (80.6%) of cases reported after the February ORV were unvaccinated. The outbreak peaked in late March 2020. Unvaccinated persons had higher odds of getting measles (adjusted odds ratio (AOR)=8.569; 95% CI [1.41- 53.4], p=0.02). Non exposed persons had a lower odd of getting measles (AOR=0.114; 95% CI [0.02-0.61], p=0.011). During 2018-2019, the accumulated number of unvaccinated children (18,587) is more than a birth cohort of the county. Persistent low routine vaccination is the most critical driver of the measles outbreaks. Low-quality ORV and the intermediate population density are secondary drivers of the outbreaks.

## Introduction

Measles is a vaccine-preventable, highly contagious disease targeted for elimination by 2020. Despite the efforts and progress made in reducing the measles burden globally, the disease was resurgent worldwide in 2018 -2019 [1]. The overriding driver for the large outbreaks was low vaccination coverage [2]. This serves to remind countries of the need to achieve and maintain high vaccination coverages for measles to be eliminated.

In 2013, South Sudan committed to the goal of eliminating measles disease in Africa by 2020 [3]. To achieve elimination, countries committed to strengthening measles vaccination to achieve a ≥95% coverage of their target populations with the first dose of measles-containing vaccine (MCV1) at national and district levels and ≥95% coverage with the measles-containing vaccine (MCV) per district during supplemental immunization activities (SIAs) [3]. Pursuant to this goal, South Sudan EPI policy requires that children <2 years be vaccinated against measles at nine months. To reduce the burden of measles, children are offered a second opportunity for measles vaccination through supplemental immunization activities (SIAs) conducted after every two years. Routine immunization services are offered through a mix of fixed, outreach, and mobile strategies. Because of logistical and security related challenges that limit accessibility to vaccination services, periodic intensification of routine immunization (PIRI) is an effective strategy for catch-up vaccination in South Sudan [4].

Despite concerted efforts by the ministry of health and partners, the coverage of routine measles vaccination has remained suboptimal in South Sudan [4, 5]. This may be contributing to the recurrent measles outbreaks in the country. In 2019, 24 counties and 4 protection of civilian camps (PoCs) reported outbreaks of measles. Repeated efforts to control the outbreaks through outbreak reactive vaccination (ORV) did not interrupt the spread of measles in some counties and PoCs. ORV is a vital strategy to bridge immunity gaps and minimize the impact of outbreaks [6]. However, logistical challenges and insufficient resources, given the short timelines, could potentially compromise the ORV and consequently prolong the outbreaks [7].

Between January 2018-March 2020, Aweil East county confirmed five measles outbreaks. Even after conducting seven outbreaks reactive vaccination (ORV) and reporting >95% administrative coverage, measles spread could not be controlled. We investigated drivers of the outbreaks and recommended probable public health interventions to interrupt the measles spread in Aweil East county.

## Methods

**Study design:** we conducted a cross-sectional descriptive study and a case control study in Aweil East county in South Sudan.

**Study area:** Aweil East is an intermediate, densely populated county in Northern Bahrel Ghazel state of South Sudan. It is situated to the north of the country bordering Sudan and has an estimated population of 487,253 with 24,363 and 102,323 children under one and ≤15 years, respectively. The county has 26 health facilities offering routine immunization services, including two faith-based facilities and two public hospitals where advanced in-patient care is provided. The county has very many hard-to-reach areas with routine immunization services. The skewed distribution of health facilities, limited cold chain capacity, and irregular outreach and mobile services contribute to the limited accessibility to RI services in this county.

**Data collection:** we reviewed the county measles line list, medical records (January-June 2020), and conducted face to face interviews with study subjects (adults who suffered measles) or guardians of children who participated in the study. Data on risk factors and the demographics of each subject were collected using a standard check list. Active case search and house to house approaches were used to search for unreported cases. Routine immunization and SIA coverage data were accessed from the county monitoring and evaluation office and verified on DHIS 2. A structured interview with study subjects (caregivers of children and adult cases) who were not vaccinated was used to identify the reasons for missing vaccination.

**Laboratory testing:** reverse transcription-polymerase chain reaction (rRT-PCR) testing of blood serum performed at national public health laboratories confirmed measles

**Study subjects:** children and adults presenting with a set of symptoms consistent with measles' case definition participated in this study.

**Case-control study:** we conducted a matched case-control study to assess potential exposures derived from the descriptive analysis. Twenty-six cases were randomly (simple random sampling) selected from among the laboratory-confirmed and epidemiologically linked cases. We matched (1:1) the cases with controls (family members of cases or neighbours of the same age group who did not have a fever, maculopapular generalized rash, and cough). A semi-structured questionnaire was used to obtain information on the demographics, date of onset, date of visit to health facilities, outcome, history of exposure to a measles case, and vaccination status.

**Data analysis:** data were entered using Microsoft Excel and analysed using the Statistical Software Package SPSS v.25 (Inc., Chicago, IL, USA). The results for categorical data are presented as frequencies and percentages. Binary logistic regression models were computed to identify the association of vaccination status, age, history of exposure to a measles case, residence, and sex with measles disease. Odds ratio (OR) and 95% CI for the regression parameters are reported. Statistical significance was set at p<0.05.

## Results

After we were notified of 3 confirmed measles cases in Aweil East county in the epidemiological week 16, we began an investigation. Health care workers were alerted of the outbreak and asked to be vigilant of more cases in the county. Using the recommended case definition for measles, active case search and line listing of cases was intensified, communities were informed of the outbreak through traditional community channels, and health workers strengthened screening of eligible children in health centres to reduce missed opportunities for vaccination. Details of measles suspected cases in the community and those presenting to facilities after the alert was issued were line-listed, but no samples were taken. The attending health worker took details of their travel in the last 21 days, the signs and symptoms, onset date, vaccination history, and contact history with a known case.

**Figure 1:**
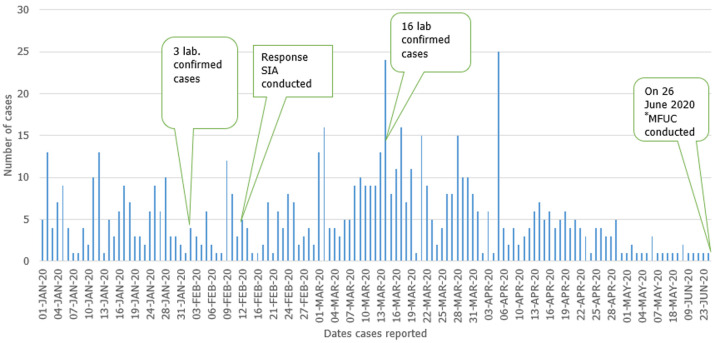
number of measles cases by date of onset of rash (n=687), Aweil East county, South Sudan, January 1-June 29^th^, 2020

**Figure 2:**
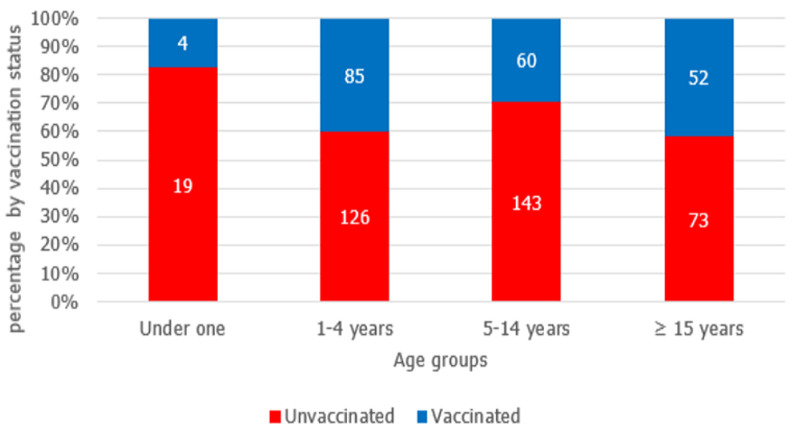
vaccination status of measles cases by age group, Aweil East, South Sudan, January-June 29^th^ 2020 (n=562); (125 under one children were not eligible for vaccination)

From January 1^st^-June 29^th^, 2020, 687 cases were reported in Aweil East county (Figure 1). The outbreak spread quickly, reaching a peak in epidemiological weeks 11 and 12. Between February 24 and March 13, 2020, a total of 16 cases were confirmed as IgM+, prompting the county to declare an outbreak. After week 12, the cases gradually declined. Among the 687 cases, 51.8% were males, and the median age was four years (range = 1 month to 70 years). About sixty-eight percent of all cases were unvaccinated. However, among persons aged nine months to 70 years, 64.2% had no history of vaccination. Although high numbers of unvaccinated were reported among 1-4 years and 5-14 years' age groups, the highest proportion of unvaccinated persons was among children aged 9-11 months (Figure 2). During January-June 2020, the rapid spread of measles was among children under five years (Table 1); overall, eight measles deaths were reported (case fatality rate = 1.16%). Cases were reported in all the Payams in Aweil East. However, the rate of spread of the disease varied among the Payams. Mangartong and Baac attack rates were higher than the county's cumulative attack rate (123 per 100,000 populations) (Table 2).

**Table 1:** age-specific attack rates of measles cases, Aweil East county, South Sudan, January-June 29^th^ 2020 (n=678)

Age category	No. of cases	population	Attack rate/100,000
< 1	141	25131	561
1-4 years	214	69809	306
5-14 years	207	167540	124
≥ 15 years	125	295988	42

**Table 2:** attack rates of measles cases by Payam, Aweil East county, South Sudan, January-June 29^th^ 2020 (n=678)

Payams	Frequency	Population	Case /100,000
Baac	293	117587	249
Madhol	40	94528	42
Malualbai	26	68907	38
Mangartong	262	84351	311
Mangok	17	52531	32
Wunlang	37	85118	43
Yargot	12	55446	22
Total	687	558468	123

**Figure 3:**
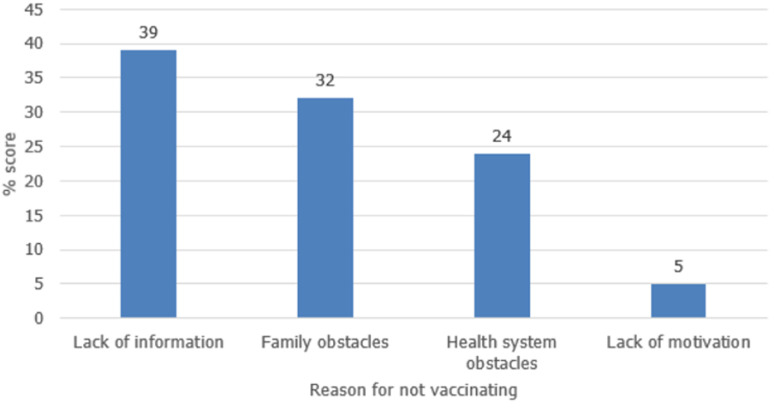
reasons for failure to vaccinate, 5 Payams´ in Aweil East, South Sudan, January-May 2020

**Routine immunization:** in 2018 and 2019 (after the last measles follow-up SIA in 2017), the annual administrative vaccination coverages were 55% and 57%, respectively. By January 2020, the county had accumulated 18,587 children (equivalent to an annual birth cohort) who were susceptible to measles (Table 3). Based on interviews conducted with cases or caregivers of the cases who were not vaccinated, lack of information regarding the importance of vaccination services, competing priorities, and long distances to health facilities are the leading causes of missing vaccination services in the county (Figure 3).

**Table 3:** number of susceptible children in Aweil East, 2018-2019 *response SIA not factored

Year	Estimated Population (< 1 year)	Vaccinated children	Unreached children	Unprotected children due to vaccine failure	*Total unprotected
2018	17,591	9,744	7,847	1,462	9,309
2019	18,118	10,400	7,718	1,560	9,278
					18,587

**Supplemental immunization activities:** between January 2018 and June 2020, 4 reactive and 1 follow-up measles supplemental immunization activities were conducted in Aweil East. The mean duration between the declaration of an outbreak and the outbreak reactive vaccination is ten weeks. However, the duration between the last outbreak in March 2020 and the measles follow-up campaign was 17 weeks due to the restriction of movements and holding meetings consequent to the COVID-19 epidemic. The follow-up SIA was not implemented in February 2020 as planned because a reactive campaign had been conducted barely two weeks prior. Even though it was reported that over 94% of children 6-59 months were vaccinated during the ORV conducted in December 2019 and February 2020, the spread of measles was not halted. Of note is that among suspected cases reported after the February SIA (between February 20^th^ to May 7^th^), 77% of children aged 1-4 had no vaccination history.

**Case-control study:** twenty-six cases were matched with 26 controls from their neighbourhood. After controlling for age, sex, and place of residence of the participants, the odd of measles among the unvaccinated persons was 8.5 times higher than the vaccinated (AOR = 8.569, 95% CI [1.41 - 53.4], p=0.02). The odd of measles among persons without a history of contact with a suspected measles' case was 0.114 compared to those exposed (AOR = 0.114, 95% CI [0.02 - 0.61], p=0.011).

**Public health responses:** surveillance for measles cases was intensified in the county. Health workers across the county were reminded of the cases' definition of measles and instructed to report all suspected cases seeking care in their facilities. County surveillance officers intensified active case search. They regularly reviewed clinical notes in health facilities, including private institutions, for missed cases, and visited schools to sensitize learners and teachers about measles.

Previously established culturally appropriate community mobilization approaches were bolstered during the outbreak. Radio messaging and person to person communication using social mobilizers, vaccinators, and other health workers, were the approaches used to pass the information on the signs and symptoms of measles and the dates and venues for the SIAs. Community mobilizers conducted house to house communities' sensitization of measles and referred suspected cases to the health facilities. With support from partners, the county developed simple job aids and messages for community mobilizers to sensitize the communities. Also, messages were aired on the radio.

In the second week of February, as the number of cases increased, the state Ministry of Health (MoH) with support from a local partner, conducted a ORV targeting children; 6-59 months in the county. Through this effort, 95% of the targeted population received a single dose of measles vaccine. Because of the short duration between the response SIA and the measles follow-up campaign (MFUC), the MFUC was deferred. One month later, the outbreak, in Aweil East, continued with the numbers of suspected cases increasing. In March 2020, 20 cases were confirmed measles IgM+. In response to the rapid increase in cases, the national EPI technical working group recommended fast-tracking the MFUC. However, because of the restrictions to movement and holding meetings occasioned by the COVID-19 pandemic, the SIA was postponed indefinitely. In June 2020, as the outbreak continued, the national EPI TWG managed to conduct an SIA under the strict observance of the COVID-19 related safety guidelines, reaching 80% of children 6-59 months of age. Three months after the SIA, no cases have been reported in the county.

Fixed and outreach EPI services were intensified during the outbreak. Cases admitted to health facilities received therapeutic vitamin A and antibiotics (for cases that presented with infectious diseases).

## Discussion

This investigative report provides a detailed description of the measles cases, probable factors related to the outbreaks, and the public health responses to the outbreaks undertaken. Between January 2019 and June 29^th^ 2020, three outbreaks were confirmed in Aweil East county after more than three laboratory cases were reported within a month. In 2020, more cases were males, and more than half of the affected persons were children below five years. Among the cases, the median age was four years, and sixty-nine percent had no vaccination history, indicating the low county´s population immunity. The high number of cases among older children and adults indicated the persistently low measles vaccination over time, increasing measles' susceptibility. Because we could not establish the index cases for the outbreak in 2020, we presumed it was a continuation of the outbreaks in 2019.

In multivariate analysis, vaccination status was the strongest indicator of susceptibility to measles. Unvaccinated persons were 8.56 times more likely to get measles than vaccinated persons. Our finding is similar to results reported in studies conducted in Ethiopia and Indonesia [8, 9]. The one-dose measles coverage in Aweil East has remained suboptimal over the last three years. In 2018, Aweil East county had the highest number of unvaccinated children in South Sudan [5]. The low coverage is thought to be caused by a combination of supply and demand-side factors. Aweil East is one of the most densely populated counties in South Sudan. Although 26 health facilities offer routine vaccination services, the facilities' spatial distribution is skewed, rendering some communities, especially those in the remotest areas, inaccessible. Outreach and mobile services to these communities have been irregular. A significant number of facilities lack cold chain equipment, hampering the provision of daily vaccination services. Low demand for vaccination services was attributed to caregivers' lack of information on vaccination services and competing priorities.

South Sudan currently provides a second opportunity for measles vaccination through supplemental immunization activities (SIAs). The last follow up SIA (before this outbreak) was conducted in 2017. In the last three years, the county has conducted seven outbreaks reactive vaccination (OVC) drives initiated and conducted by the state ministry of health and its partners. Of note is that despite the reported high administrative vaccination coverage, the OVCs did not stop the spread of the disease, probably due to the suboptimal quality of the SIAs. This challenge is best illustrated by the low proportion of cases reported after the SIA that had a vaccination history. Unlike planning for preventive SIA that takes 12-15 months [10], ORV should be conducted soon after confirmation of an outbreak to interrupt the spread and minimize mortality [11]. The limited-time to adequately plan, coupled with insufficient resources, impacts the quality of the OVC. Furthermore, a delayed response in an intermediate densely populated area prolongs the duration of the outbreak, as witnessed in Aweil East between March and June 2020 [7].

Measles is a communicable and highly contagious disease, with an average secondary attack rate of 52-90% [12]. A single case of measles could infect up to 18 unimmunized persons [13]. In multivariate analysis, exposure to a measles case was associated with significantly increased odds of measles. The cumulative attack rate was higher than in studies in Uganda, Ethiopia, states in Nigeria's southern region, indicating a delayed response to the outbreaks [14-16]. Also, the rapid spread was aided by the low population immunity in this county. The variation in the attack rates by Payams could reflect the disparities in vaccination coverage and the skewed spatial distribution of facilities affecting the reporting of cases. The measles risk was highest among children under five years, indicating a persistent low vaccination coverage in the past four years. The low attack rate among persons above 14 years may indicate protection from the natural disease and, to a lesser extent, vaccination. We found an overall case fatality rate of 1.16%. Because of the low access to health care in the county, deaths in the community were most likely never reported to the health system. We, therefore, consider that the reported fatality rate could be an underestimate of the actual figure. Studies conducted in Nigeria, Ethiopia, and Guinea reported a lower CFR [9, 14, 16, 17].

Although many children aged <9months were listed as suspected cases of measles, the youngest person reported with lab-confirmed measles was seven months. The low numbers may reflect the passive immunity from maternal antibodies known to persist in the first year of life. We inferred that the low attack rate among persons of 14 years, which indicated that most child-bearing age women were protected from measles and conferred passive immunity to their infants. However, the fewer samples taken from younger children, possibly due to the difficulties in collecting samples from this age group, could have affected the overall results.

We acknowledge the following limitations of this study that the reader should consider when interpreting the findings: numerous cases in this study were epi-linked to confirmed cases and not through laboratory testing; vaccination status of many cases were confirmed through recall as vaccination card retention was low and health facility vaccination records were found to be unreliable or missing especially for older children and because of the low number of cases in the analytical study, this study did not assess the effectiveness of the measles vaccination. Future studies in South Sudan should establish the effectiveness of the measles vaccination.

## Conclusion

Persistent low routine vaccination is the most critical driver of the measles outbreaks. The low community awareness on the importance of immunization services coupled with suboptimal accessibility to vaccination services in Aweil East are contributors to the low vaccination coverage.
